# Maternal Supplementation with Cow’s Milk Naturally Enriched with PUFA Alters the Metabolism of Sows and the Fatty Acid Profile of the Offspring

**DOI:** 10.3390/nu13061942

**Published:** 2021-06-05

**Authors:** Leriana Garcia Reis, Thiago Henrique Silva, Gisele Mouro Ravagnani, Cristian Hernando Garcia Martinez, Márcia Saladini Vieira Salles, André Furugen Cesar Andrade, Nara Regina Brandão Cônsolo, Simone Maria Massami Kitamura Martins, Fernando de Oliveira Bussiman, Mauricio Xavier Silva Oliveira, Dante Pazzanese Duarte Lanna, Arlindo Saran Netto

**Affiliations:** 1 Department of Animal Science, School of Animal Science and Food Engineering, University of São Paulo, Avenida Duque de Caxias Norte, 225, Pirassununga 13635-900, Brazil; leriana@usp.br (L.G.R.); silvath@usp.br (T.H.S.); nara.consolo@hotmail.com (N.R.B.C.); smmkm@usp.br (S.M.M.K.M.); 2 Department of Animal Reproduction, School of Veterinary and Animal Science, University of São Paulo, Avenida Duque de Caxias Norte, 225, Pirassununga 13635-900, Brazil; giselemouro@gmail.com (G.M.R.); andrefc@usp.br (A.F.C.A.); 3 Department of Animal Nutrition and Production, School of Veterinary and Animal Science, University of São Paulo, Avenida Duque de Caxias Norte, 225, Pirassununga 13635-900, Brazil; chmartinez.mvz@usp.br (C.H.G.M.); fernando.bussiman@usp.br (F.d.O.B.); 4 Animal Science Institute, Avenida Bandeirantes, 2419, Ribeirão Preto 14030-640, Brazil; marcia.saladini@gmail.com; 5 Department of Animal Science, Ohio Agricultural Research and Development Center, The Ohio State University, Wooster, OH 44691, USA; oliveira.51@osu.edu; 6 Department of Animal Science, Luiz de Queiroz College Agriculture, University of São Paulo, Avenida Pádua Dias, 11, Piracicaba 13418-900, Brazil; dplanna@usp.br

**Keywords:** cholesterol, edible oil, fatty acid metabolism, n-3 fatty acid, n-6 fatty acid, polyunsaturated fatty acid

## Abstract

The study aimed to evaluate the supplementation of gilts with cow’s milk naturally enriched with n-3 and n-6 polyunsaturated fatty acids (PUFA) on reproductive outcomes, and the serum biochemical and FA profile of swine females and their offspring. During 316 days, 30 gilts were distributed into three groups: (1) Control, fed a basal diet + milk from cows without oil; (2) n-3, fed a basal diet + milk from cows fed a diet enriched with linseed oil; (3) n-6, fed a basal diet + milk from cows fed a diet enriched with soybean oil. The gilts receiving the diets containing PUFA had higher serum urea and very-low-density lipoprotein levels and lower serum total protein and low-density lipoprotein levels compared to the Control group. Females supplemented with n-3 presented higher serum palmitic acid and γ-linolenic acid levels than those fed n-6. Piglets from the Control group were heavier at birth than those from females supplemented with enriched milk. The piglets from females receiving enriched milk had 140 g higher body weight from 1 to 21 days old compared to the Control group, and greater average daily weight gain from 7 to 14 days old. The serum eicosapentaenoic acid level of piglets fed n-3 was 69% higher than those fed n-6, which reduced the AA/EPA ratio. Gilts supplemented with PUFA-enriched cow’s milk showed changes in their serum palmitic and γ-linolenic acid levels, in addition to improved performance, EPA concentration and consequently reduced AA/EPA ratio in their piglets, demonstrating beneficial results for their progeny.

## 1. Introduction

Polyunsaturated fatty acids (PUFA) have been emphasised as bioactive compounds correlated with cardiovascular health by reducing the levels of risk factors such as cholesterol level, reduced risk of diabetes and improved human health [1,2]. Maternal supplementation with PUFA may be an essential source of those fatty acids (FA) for foetal and newborn development [3,4], mainly because PUFA can be a precursor of long-chain polyunsaturated fatty acids (LC-PUFA) such as docosahexaenoic acid (DHA) and eicosapentaenoic acid (EPA), which are essential for the development of foetal and infant vision, brain, immune system and inflammatory response and to protect against some allergies and disease [5].

Maternal supplementation can be by the intake of foods with content of those compounds. In this case, milk and dairy products make an important contribution to consumption of essential FA, especially because they are products consumed daily, and also because their composition can be manipulated through the animal’s nutrition [6,7]. Studies have shown that PUFA supplementation during gestation and lactation can alter serum the FA profile of neonates, such as of humans [3], dogs [8], sheep [9], goats [10], rats [11] and pigs [12,13]; however, there are no long-term studies using cow milk as a natural source of PUFA.

Animal models are used extensively in nutritional research. The suitability of the pig (*Sus scrofa*) as a model for human research is becoming accepted, particularly because humans and pigs are classified as omnivorous mammals and share similarities related to anatomical features of the gastrointestinal tract [14]. In this sense, the supplementation of swine with enriched cow milk could be used as a model for human nutrition, providing a deeper understanding about its effect on human nutrition and metabolism. Therefore, we hypothesised that gilts supplemented with cow milk enriched with PUFA would improve their health, as well as the health and performance of their piglets. The objective of this study was to evaluate the effects of supplementation of gilts with cow’s milk enriched with n-3 and n-6 on maternal blood metabolic profile, sow’s reproductive outcomes, colostrum and milk composition, colostrum and milk FA profile, piglets’ performance and the serum FA profile of both.

## 2. Materials and Methods

The trial was performed in the Swine Research Laboratory of the School of Veterinary Medicine and Animal Science (FMVZ), University of São Paulo. The procedures and experimental use of animals were approved by the Animal Care and Use Committee of the School of Animal Science and Food Engineering at the University of São Paulo (Protocol #4939070317).

### 2.1. Experimental Design

In this case, 30 hybrid gilts (Landrace x Large White), 34 days old and initial body weight of 9.59 ± 1.28 kg until first farrowing, were housed individually and used in a completely randomised design experiment. Gilts were randomly allocated into one of three groups: (1) Control-basal diet + milk from cows without oil supplementation; (2) n-3—basal diet + milk from cows fed a diet enriched with linseed oil; and (3) n-6—basal diet + milk from cows fed a diet enriched with soybean oil. The gilts were supplemented with the treatments until their offspring were 21 days old (316 days). The enriched milk was obtained from Holstein cows from the University of São Paulo herd, more details can be find in the study of Oliveira et al. [15]. The cows were supplemented or not with 2.5% (on a dry matter (DM) basis) of linseed or soybean oil, sources of n-3 and n-6, respectively.

The effects of vegetable oil on the quality of the lipid fraction of the dairy cows’ milk provided for gilts are presented in Table 1. From weaning to artificial insemination (AI), the females were supplemented with 200 mL (days 34–76), 300 mL (days 77–128), 400 mL (days 129–174) and 500 mL (days 175–247) of cows’ milk per animal per day (Supplementary Figure S1). From oestrus synchronisation (Regumate^®^, MSD Saúde Animal, São Paulo, Brazil) up to the end of the lactation period, females received 1 L/day of cows’ milk. Supplementation with milk was performed daily at 8:00 a.m., after individual feeding.

### 2.2. Diets

Water and feed were provided ad libitum and feed was offered according to the phases described below: pre-starter phase, from weaning to 43 days old; starter phase, from 44–72 days old; grower phase, from 73–130 days old; finisher phase, from 131–152 days old; replacement phase, from 155–AI; gestation phase; and lactation. The basal diet was formulated to meet the nutritional requirements of females for each physiological phase according to Rostagno et al. [16]. All diets were based on corn and soybean feedstuffs and did not contain any oil as an ingredient (Table 2). The gilts were fed twice a day, at 7:00 am and 1:00 pm. The descriptive analysis of the FA profile and lipid fraction of diets is shown in Table 3.

### 2.3. Management of Swine Females and Data Collection

After synchronisation, gilts were checked for standing oestrus by placing a entire male pig into the pen for 15 min/day. Gilts were inseminated with pooled semen every 24 h until the end of the oestrus. Approximately 21 days after AI, pregnancy was detected using a real-time ultrasound (Scanner 100^®^, 5 MHz transducer, Pie Medical, Maastricht, The Netherlands). At approximately 107 days of pregnancy, gilts were transferred to individual farrowing crates built of concrete and equipped with slatted floor and an infrared lamp to establish a warm microclimate for the piglets.

All females were closely monitored during farrowing starting from the birth of the first piglet until placenta expulsion. After farrowing, no creep feed was offered. For each litter, the numbers born alive, stillborn or mummified and total piglets born were recorded along with the time of birth and birth weight for each piglet, females were monitored until day 21 of lactation. Colostrum samples (20 mL) were collected in sterile tubes by manual expression from multiple teats at the moment milk ejection started during farrowing, and milk samples (50 mL) were collected 14 d post-farrowing after injection with 1 mL oxytocin (Ocitovet, Ceva, Brazil) in the ear vein. Colostrum and milk samples were immediately frozen at −20 °C until later analysis. The milk composition was analysed using an automatic milk analyser (Lactoscan MCC, Nova Zagora, Bulgaria).

The FA profile of diets, colostrum and sow milk was determined by the Animal Nutrition and Growth Laboratory of the Luiz de Queiroz School of Agriculture (ESALQ/USP). Feed samples were collected and approximately 80 mg of each was ground on a 1-mm screen mill and transferred to an extraction tube (10 × 1.4 cm), to determine the composition of FA offered. Then, 2 mL of methanol/acetyl chloride (20:1) was added along with 1 mL of hexane and the solution placed in a water bath at 90 °C for 10 min. After cooling to ambient temperature, 2 mL of distilled water was added and centrifuged at 3200 rpm for 5 min. The supernatant liquid was transferred to a chromatography bottle; this method was adapted from Rodríguez-Ruiz et al. [17]. The colostrum and milk samples were defrosted and centrifuged at 4700 rpm for 45 min. Then, 400 mg of fat was removed from the supernatant for fat extraction according to Hara and Radin [18]. Methylation was by a methanolic sodium methoxide solution, according to Christie [19]. The FA profile was determined by gas chromatography (ThermoFinnigan^®^, model Trace 2000, Thermo Fisher Scientific, Waltham, MA, USA), using a fused sílica capillary column, CP-Sil 88 (100 mm × 0.25 mm × 0.2 mm; Agilent Technologies, Ankeny, IA, USA). Hydrogen was used as carrier gas at a flow rate of 1.8 mL/min, and vaporiser and detector temperatures were 250 and 300 °C, respectively. The oven temperature programme was initially 70 °C with a holding time of 4 min, then 175 °C (13 °C/min) with a holding time of 27 min, 215 °C (4 °C/min) with a holding time of 9 min and finally an increase by 7 °C/min to 230 °C and standing for 5 min. A 2 μL aliquot of the esterified extract was injected into the chromatograph and the identification of FA was performed by comparing the retention times and the percentages of FA using the software Chromquest 4.1 (Thermo Electron, Thermo Fisher Scientific, Waltham, MA, USA). Standards used were Supelco 37 Component FAME Mix (CRM47885, Supelco, St. Louis, MO, USA) and linoleic acid (conjugated methyl ester, O5632, Sigma-Aldrich, St. Louis, MO, USA). The FA concentration was expressed in g/100 g of fatty acid methyl esters (FAME).

### 2.4. Sows’ Performance and Profile of Metabolite in Serum

Sows were weighed on the 1st and 21st day of lactation after the morning feed. The blood samples were collected from a jugular vein on the first day of lactation after morning feeding. Samples were centrifuged at 2000× *g* at ~25 °C for 10 min and the serum stored at –20 °C. The following assays were performed to determine the metabolic profile: total cholesterol, high-density lipoprotein (HDL), total protein and serum urea using enzyme kits (VIDA Biotecnologia^®^, Minas Gerais, Brazil). Low-density lipoproteins (LDL) and triacylglycerol were analysed using enzyme kits (LABTEST^®^, Minas Gerais, Brazil) and the serum FA profile was analysed by gas chromatography [20]. The very-low-density lipoprotein (VLDL) concentration was determined indirectly by the following equation [21]: VLDL cholesterol (mg/dL) = triglyceride concentration/5. The chemical analyses were determined in the Clinical Analysis Diagnostics laboratory (DAC, Pirassununga, SP, Brazil) using the specific assay kits mentioned above according to the manufacturer’s instructions. 

### 2.5. Management of Piglets and Data Collection

Routine procedures (teeth clipping, tail docking, ear notching and iron injection) were conducted 2 days after farrowing, and no creep feed was offered. The piglets were weighed at birth before the first suckling and at 7, 14 and 21 days old and average daily gain (ADG) was measured. The basal diet was provided from d 14 of old up to d 21 when the piglets were weaned. Both water and feed were offered ad libitum. Blood samples were collected at birth (before the first suckling) and at 14 days old by jugular vein puncture into an amber tube without EDTA for analysis of the serum FA profile by gas chromatography [20].

### 2.6. Chemical Analysis of Diets

The nutritional levels of diets were analysed in duplicate according to the rules of the Official Association of Analytical Chemists [22]. Analyses of DM (method 930.15), ashes (method 942.05), crude energy (EB), total phosphorus (P, method 946.05), calcium (Ca, method 978.02) and nitrogen (N) was made using the Kjeldahl digestion and distillation procedure (TE-036/1, Tecnal, Brazil) and the crude protein was calculated as N × 6.25 (method 990.03), and crude fibre (CF) [23]. The ether extract (EE) was analysed in duplicate according to the rules of the Official Association of Analytical Chemists [24].

Lipids were extracted from samples of the diets according to the Folch method using chloroform/methanol at 2:1 *v*/*v* [25]. A sample of approximately 1 g was used to determine the FA composition of the diets offered. A frozen sample was homogenised in 20 mL of a solution of chloroform/methanol (2:1) using a Turrax homogeniser, disintegrator and emulsifier. After that, an aliquot of lipid extract was methylated according to [26]. The quantification of lipids used was by GC 2010 gas chromatograph (Shimadzu Corp., Kyoto, Japan) with a SP-2560 capillary column (100 mL × 0.20 mm D with 0.02 μm film thickness; Supelco, Bellefonte, PA, USA). Initially the temperature was adjusted to 70 °C for 4 min and then increased by 13 °C/min until reaching 175 °C and then maintained for 27 min. The temperature was then increased by 4 °C/min to 215 °C and then maintained for 31 min. Hydrogen was used as carrier gas at a flow rate of 40 cm^3^/s.

### 2.7. Statistical Analyses

All statistical analyses were performed using SAS version 9.4 (SAS Inst. Inc., Cary, NC, USA). The data were analysed in a completely randomised design, and the animal was considered as an experimental unit. The animals were distributed randomly into one of three treatments. The normality of the residuals was verified by the Shapiro-Wilk test (PROC UNIVARIATE from SAS) and information with studentized residuals greater than +3 or less than −3 were excluded from the analyses. The Levene test compared the homogeneity of the variances. Variables with continuous distribution were analysed using the SAS MIXED procedure (SAS Institute Inc.). When the residuals did not follow a gaussian distribution, log transformation was applied and if the residuals remained not being normal distributed a generalized linear model was used with Log-Normal distribution. When the time factor was not present, the statistical model included ‘treatment’ as a fixed effect, ‘animal’ and ‘residual’ as random effects. When the time factor was present, repeated measures in time were performed, in which the statistical model included the fixed effects of ‘treatment’, ‘time’ and ‘treatment by time interaction’. The ‘animal effect’ and ‘residual’ were considered to be random. The discrete variables, such as total number of piglets born per litter, number of piglets born alive per litter, number of stillbirths per litter, number of mummifieds per litter and time to reach the ceiling were analyzed using the GLIMMIX procedure, Poisson distribution. The Kenward-Roger method was used to approximate the degree of freedom of the denominator for the F-test in statistical models. The covariance structure for each parameter was determined based on the lowest value of the Akaike information criteria. In addition, data from the first measurement of weight and weight on the day of delivery were used as covariates in statistical statistics when appropriate. The treatments were analyzed as orthogonal contrast, with contrast 1 (C1): Control group vs. n-3+n-6, and contrast 2 (C2): n-3 vs. n-6. The differences were considered significant at *p* < 0.05, and a trend was considered when 0.05 < *p* ≤ 0.10.

## 3. Results

### 3.1. Sows’ Performance, Serum Metabolite Profile and Colostrum FA Profile

No differences were found in the average body weight of the females during lactation (Control 179.29 kg; n-3 183.29 kg; n-6 180.62 kg; Supplementary Table S1). The highest percentage of body weight loss was observed for the Control group (9.42%), n-3 (6.48%) and n-6 (7.92%) during lactation (d 1–21; *p* > 0.05). This weight difference was greater by 70.22% and 49% for the Control group and n-6 compared to n-3 (*p* > 0.05), respectively. The n-3 group presented a gain of 5.27% in backfat thickness, while in the other groups losses were observed (2.76% in Control group and 4.18% in n-6) from d 1–21 (*p* > 0.05). Based on n-3 females, the Control and n-6 groups showed losses of 158.62% and 185.06%, respectively (*p* > 0.05), in backfat thickness (Supplementary Table S1).

On the first day of lactation, females supplemented with milk enriched with n-3 and n-6 presented higher serum concentration of urea (*p* < 0.001) and VLDL (*p* = 0.043), and lower total protein (*p* = 0.010) and LDL (*p* = 0.012) concentrations (Table 4). There was a trend to increase serum triglyceride concentration (*p* = 0.062) in sows fed n-3 and n-6 compared to the Control (Table 4). However, no differences (*p* < 0.100) were found for total serum cholesterol, glucose or HDL concentrations among treatments, and there was no difference in serum biochemical parameters between treatments n-3 and n-6 (Table 4).

According to the sows’ serum FA profile on the first day of lactation (Table 5), females supplemented with n-3-enriched milk had 17.58% higher palmitic acid (16:0; *p* = 0.012) and 47.92% higher γ-linolenic acid (18:3n6; *p* = 0.031) compared to n-6 sows. For stearic acid, sows fed n-3 had a tendency towards lower concentration than those fed n-6 (18:0; *p* = 0.066, Table 5). Furthermore, females that received enriched milk tended to show decreased serum linoleic acid (18:2n-6; *p* = 0.079), PUFA (*p* = 0.084) and total n-6 (*p* = 0.081) when compared to the Control group (Table 5). Colostrum from females supplemented with enriched milk tended to have lower protein (*p* = 0.075) and lactose values (*p* = 0.087) compared to the Control group (Table 6) on day 14 of lactation. The other variables of serum FA profile and colostrum composition were not different among treatments (*p* > 0.05).

Sows fed the Control diet had higher concentrations (*p* < 0.05) of isotridecyl (13:0 iso), isomargaric (17:0 iso) and n-6/n-3 ratio and lower concentrations of isopentadecylic (15:0 iso), cis-10-Heptadecenoic (17:1c10), elaidic (18:1t), cis-15-Octadecenoic (18:1c15), α-Linolenic (18:3 n-3) and Σn-3 at d 1 of lactation compared to those fed n-3 and n-6 diets (Table 7 and Table 8). The treatment was significant (*p* < 0.05) for isopentadecylic (15:0 iso), isomargaric (17:0 iso), elaidic (18:1t), cis-15-Octadecenoic (18:1c15), α-Linolenic (18:3 n-3), Σn-3 and n-6/n-3 ratio (Table 7 and Table 8). Sows supplemented with milk enriched with linseed oil showed an increased (*p* < 0.05) cis-15-Octadecenoic (18:1c15), α-Linolenic (18:3 n-3), EPA (20:5 n-3) and Σn-3 and a decreased of pentadecylic (15:0) and n-6/n-3 ratios in their colostrum, compared to the n-6 group (Table 7 and Table 8).

Consequently, for the FA profile of sows’ milk sampled at 14th day of lactation, the treatment was significant (*p* < 0.05) and the sows in group n-3 had a higher concentration (*p* = 0.001) of cis-15-Octadecenoic (18:1c15) compared to the n-6 group, but lower concentration of ARA (20:4n-6; *p* = 0.012) compared to the sows fed n-3 (Table 9). Additionally, sows fed the n-3 diet presented a lower (*p* = 0.026) n-6/n-3 ratio in milk at d 14 of lactation compared to those fed the n-6 diet (Table 10).

### 3.2. Piglets Performance and Profile of Metabolite in Serum

Piglets fed the Control diet had shorter time to first suckle (*p* < 0.001) than the other groups, and the n-3 group also presented lower time (*p* < 0.001) compared to the n-6 animals. There were no differences in the numbers of total piglets born, born alive, stillborn or mummified per litter (*p* > 0.05, Table 11). Piglets born to females supplemented with enriched milk had a lower birth weight (*p* < 0.001), an increase of 140 g in the total weight from d 1–21 (*p* = 0.004) and greater weight gains from d 7–14 by 18.75% (*p* = 0.003, Table 11) compared to the Control group.

Piglets born from sows supplemented with n-6 milk had higher weight at birth (*p* < 0.001) and at d 21 after birth (*p* = 0.035) than piglets born from the n-3 group (Table 11). The average weight gain from d 1–7, and from d 14–21, were not different among treatments (*p* > 0.05). As expected, there was an increase in body weight and weight gain for piglets over time (*p* < 0.001) where body weight increased from 1.2167 kg to 4.5533 kg, and weight gain from 0.1400 kg to 0.1567 kg, regardless treatment. Piglets from n-3 and n-6 treatments had greater body weight gain from 1 to 21 days of age compared to the Control group. As a consequence, from the age of 1 to 21 days, there was an effect for time (*p* < 0.001; Table 11).

There were several changes in piglets’ FA serum concentration according to the treatments and over time (*p* < 0.05, Table 12), in which most of the FA decreased from 1 to 14 days age, except linoleic, α-linolenic, unsaturated, polyunsaturated, total n-6 fatty acid and the ratio n-6/n-3, which increased over time. The piglets from sows supplemented with milk enriched with n-3 had a 69% higher serum EPA (20:5 n-3) concentration than those fed n-6 (*p* = 0.010). Consequently, the ARA/EPA ration was also decreased (*p* = 0.045) in the n-3 group compared to the n-6 (Table 12).

## 4. Discussion

Supplementation with milk enriched with n-3 and n-6 resulted in significant changes in the FA profile of sows and their offspring, as well as piglet performance. Other studies have also shown that the FA composition of the diet of sows affects milk composition and the performance of piglets [4,27,28]; however, there are no studies using naturally enriched cow’s milk as a long-term source of PUFA as a model for application in humans.

Females’ body weight loss was observed in all treatments over time, which was expected, since in the lactation period there is a high requirement for milk production associated with inadequate feed intake, when the females begin intense catabolism [29]. This is very similar to what happens in women during breastfeeding, because maternal energy metabolism during human lactation is characterised by augmented glucose production and increased mobilisation of fat from maternal deposits [30].

The serum biochemistry at d 1 of lactation presented several changes in the metabolites between control and omega groups. According to protein metabolism, the serum urea increased, and the total protein decreased in sows supplemented with n-3 and n-6 at d 1 of lactation. It is already reported that n-3 can play an anabolic role and improve muscle mass by reducing the total available protein [31]. Additionally, it is known that n-3 has anti-inflammatory properties, which can also be an important reason for its supplementation during female pregnancy and lactation, since immunological issues are one of many challenges that the female encounters after farrowing.

The balance of the immune system, which involves metabolic processes such as the regulation of cytokines, can alter blood levels of total protein when supplemented with different lipid profiles. PUFA supplementation may be able to reduce certain inflammatory proteins [32] and increase other anti-inflammatory agents, just as SFA can contribute to a greater inflammatory state and increase the concentration of pro-inflammatory cytokines in the blood [33]. Even so, interpretation of the differences between treatments is very complicated because the immediate post-farrowing period is followed by changes in glomerular filtration rate, DM intake and muscle mass, which may have variable effects on blood metabolites [34].

Cholesterol metabolism was also influenced by the sows’ supplementation, where the females fed with n-3 and n-6 diets presented higher levels of VLDL compared to those fed a Control diet. This result can be explained by the lipid metabolism in the small intestine, since long-chain FA are transported in the form of TG by VLDL (the main carrier). It is also important to remember that in the final third of pregnancy there are usually reductions in FA synthesis and LPL activity and an increase in triglycerides and insulin resistance. This lipolysis leads to an accumulation of lipolytic products in the main destination, the liver. Therefore, in this context it is normal to find higher values for VLDL and LDL, since maternal hyperlipidaemia results in an increase mainly in TG-rich lipoproteins, which transport LC-PUFA [35].

A 22% reduction in serum LDL-cholesterol levels in sows supplemented with enriched cow milk may be associated with the lower saturated and higher unsaturated FA content in that diet compared to that of the Control group. Some saturated FA of chain length 10–18 carbons were reported to elevate levels of mRNA para PGC-1β, which activates the family of SREBPs and regulates at least 30 genes involved in the synthesis and metabolism of cholesterol, including genes for LDL [36]. In the same way, others suggest that a great amount of linoleic acid in a diet can decrease blood cholesterol and LDL-cholesterol concentrations, particularly when it replaces the common saturated FA [37]. Consequently, the present results suggest that the milk enriched with PUFA may have a beneficial effect on maternal health.

Complementing the effect of enriched milk on lipid metabolism, serum triglyceride concentrations tended to increase in females supplemented with n-3 and n-6 relative to the Control group. According to Azain [38], triglycerides in sow serum can be considered as an index of survival for piglets because the triglycerides are metabolised rapidly to ketone bodies, can readily cross the placenta and are used in the developing foetus for the synthesis of lipids and to spare glucose. In this sense, ketogenic agents have the potential to improve foetal energy stores at birth, and consequently, improve the rate of survival [38].

At the end of pregnancy, foetal growth is maximum, the requirements for LC-PUFA are improved and the lipoprotein receptors present in the placenta capture these molecules and diffuse into the foetal plasma [39]. Little glycerol is transported, as the number of ketone bodies present can be important in conditions of maternal hyperketonaemia, and the demand for cholesterol by the foetus is high. However, what satisfies their needs is the biosynthesis of foetal cholesterol since maternal cholesterol contributes substantially to this [39].

This indicates that supplementation of sows with enriched cow’s milk may contribute to improved piglet development. However, despite those changes in serum biochemistry at d 1 of lactation verified in our study, all those parameters are within the normal range for the lactation period, as previously described by [34]. Results contradictory to the present study were reported by [40] where the administration of cow’s milk enriched with PUFA as a supplement to rats resulted in increased serum HDL and a reduction in triglyceride, VLDL and LDL levels.

Additionally, at d 1 of lactation, there were some differences in serum FA profile among the treatments. Sows fed n-3 presented higher levels of palmitic acid and a tendency for lower levels of stearic acid compared to those fed n-6, which may be due to the de novo synthesis of FA. The biosynthesis of FA occurs in the cytosol from the addition of acetyl-CoA in the form of malonyl-CoA, and the main FA produced is palmitic acid [36,41]. In the endoplasmic reticulum and mitochondria, this acid can be elongated to synthesise stearic acid. Both can be precursors of palmitoleic and oleic MUFA, common in animal tissues. It is suggested that the n-3 treatment favoured a greater availability of acetyl-CoA in the cytosol for the higher formation of palmitic acid, and the n-6 acted on the endoplasmic reticulum, favouring the formation of malonyl-CoA and NAPDH, or on the mitochondria to promote the synthesis of acetyl-CoA and NADPH as substrates for stearic acid [36,41].

The FA supplementation is known to alter the expression of some genes for the desaturase enzymes responsible for synthesising palmitoleic and oleic acids from palmitic and stearic acids, respectively [36,41]. Despite no statistical effect for oleic acid, sows fed with n-3 presented 27% higher values compared to the n-6 group. Additionally, a tendency to decrease linoleic acid (18:2 n-6) in sows supplemented with enriched milk might be attributed to the use of this FA to generate EPA and DHA. Sows fed n-3 and n-6 had 10.34% higher EPA and 32.50% higher DHA compared to the Control group. As explained above, PUFA can modulate the expression of the genes for desaturase enzymes that convert linoleic and linolenic acids to EPA, DPAn-3 and DHA [36]. Furthermore, the higher concentration of serum γ-linolenic acid (18:3 n6) in the n-3 group compared to n-6 may also have occurred by modulating the expression of genes for the enzymes presented above.

Fritsche, Huang and Cassity [42] fed sows with fish oil or a Control diet and found that the content of n-3 PUFA in the serum of sows fed with linseed oil increased six-fold over that in serum of sows fed with a Control diet; also, levels of arachidonic acid in maternal serum increased by approximately 50%, and similar changes were reflected on the FA profiles of sows’ milk. Similarly, [43] studied sows fed with linseed or linseed oil during the last third of gestation and found increases in PUFA and n-3 FA and a decrease in the n-6/n-3 ratio in the milk and blood of sows and in carcasses of newborn piglets.

Even though there were no changes in the n-6/n-3 ratio in sow’s serum among treatments, animals fed n-3 and n-6 diets had a lower n-6/n-3 ratio in colostrum (d 1 of lactation) and sow’s milk (d 14 of lactation) compared to the Control group, similar to the results of [43]. These changes in colostrum are mainly due to the linseed oil added to the cows’ diets. Even though the ARA concentration was higher in the n-6 group, this result is due to the better-quality PUFA profile. The individual contents of each family of omega-FA are extremely relevant, but when it comes to the metabolism and roles of FA, the total n-6/n-3 balance may be more relevant than the individual contents, as we can see in this case.

So far, the results of the present study showed that PUFA supplementation to sows may not only improve the health parameters of the sow but also improve the colostrum and milk FA profiles, providing better-quality feeding to the piglets (greater amounts of PUFA), which in turn can improve their survival rate and health. The essential FA are fundamental to several functions in the body, such as maturation and activities of the nervous system [44], modulation of the immune system and allergic diseases [45], maturation of the visual system [46] and cognitive and motor development [47]. It is relevant to understand the effects of these FA, since AA and DHA accumulate in the brain during the initial period of postnatal development when milk represents the only source of fat [48].

In the present study, no differences were noted among treatments for the total number of piglets born, born alive or stillborn; however, the n-3 group had 1.34 more piglets than the Control group. Moreover, when we look at the data of the percentage of live piglets born per litter, we can find 91% for the Control group, 95% for the n-3 group and 90% for the n-6 group. Mateo et al. [49] supplemented gilts with milk enriched with n-3 and reported no differences among groups in the total number of piglets born, born alive or stillborn. However, there are studies in humans and animals that report the positive effects of PUFA supplementation on reproductive aspects such as improvements in embryo morphology and pregnancy rates. They attribute this positive impact on fertility as likely to include improved oocyte quality, embryo implantation, reproductive hormones or cycle functioning [50,51,52].

Additionally, the shorter time to first suckling by piglets in the Control group can be correlated with higher newborn body weight (BW) of this group, since it is generally acknowledged that heavier piglets have more rapid access to the teats [53]. However, it did not play an essential role in this study in the BW at 21 days and the ADG, because from d 0–21, the piglets from sows supplemented with enriched milk increased their BW by 140 g more than those in the Control group. The higher ADG for n-3 and n-6 groups from d 7–14 compared to the Control group occurred because PUFA are critical to the formation of tissues and regulation of the immune functions, and they are brain lipid components, having significant effects on brain development and function and, thus, behaviour [54]. Even though there were differences in body weight of the newborns, all the results were normal, as expected. These results might also be indicative that the supply of PUFA-enriched milk can improve the energy supply from d 7–14 of life for piglets.

Supplementation with PUFA n-3 has been an effective strategy to reduce the incidence of premature birth, and also to decrease the number of babies born with low weight [55]. In the present study the groups supplemented with milk enriched with n-3 and n-6 presented better weight gain, and this can benefit babies born prematurely, since they are usually born lighter. Harauma et al. [56], in their study with rats, showed that a proper balance between PUFA sources can provide better weight gain.

Rooke et al. [57] also suggested that the increase in growth in piglets from sows fed with a diet enriched with salmon oil was a consequence of improved piglet health status (i.e., brain FA composition). Similar to the present data, [49] reported no differences in initial piglet BW according to treatments, but at d 10 and d 21 of lactation, the BW of piglets from sows fed with n-3 were higher than the Control group. Rooke et al. [57] reported that sows fed with diets enriched with n-3 produced lighter pigs at birth, but these piglets had a higher pre-weaning survival rate than the Control group. Other studies also reported indications that supplementation with n-3 and n-6 FA in the diet of gilts can benefit pregnant and lactating sows under catabolic conditions, improving foetal growth, neonatal health and lactation performance [58,59].

It was recently shown that the FA profile of the plasma and tissues of suckling piglets is highly dependent on the maternal dietary composition, and these results persist for up to 3 weeks after weaning [12]. In the present study, the milk enriched with n-3 supplemented to pregnant and lactating sows resulted in a significant increase in the serum EPA of piglets by 90.91% compared to the n-6 group, from d 0–14. Consequently, the ARA/EPA ratio was lower for milk enriched with n-3, due to the higher amount of α-linolenic acid in the maternal n-3 group, which must be converted to a 20-carbon FA, EPA (20:5 n-3) and a 22-carbon FA, DHA (22:6 n-3) through Δ6-desaturase, Δ5-desaturase and elongase enzyme metabolic pathways [5,36]. Even though we found a higher concentration of EPA in colostrum, the increase of this FA in the blood of piglets was unrelated, since collection was carried out in the piglets before suckling.

EPA is known to be crucial for healthy neural and brain growth and development [5]. In our results we saw a significant increase in EPA in piglets’ serum FA, demonstrating that the sows supplemented with milk enriched with n-3 can improve piglet development. These results also suggested that the FA composition of piglets’ serum under a maternal diet supplemented with enriched cow’s milk might be attributed not only to the incorporation of these fatty acids from the diet, but also to possible activation of their biosynthesis in tissues.

Brazle et al. [60] reported an increase in n-3 LC-PUFA in the placenta of sows fed with α-linolenic acid-rich diet during gestation, suggesting the synthesis of n-3 LC-PUFA from dietary α-linolenic acid, which can be transferred to the piglets through the placenta. This is similar to that observed in human subjects, where n-3 LC-PUFA are transferred from mother to foetus by a specific placenta transporter called plasma membrane fatty acid-binding protein [61]. Quelen, Boudry and Mourot [28] also found that the maternal diet influenced the FA composition of piglet tissues. Piglets born from sows fed with linseed oil presented higher proportions of n-3 LC-PUFA than those born from sows on a basal diet, whereas in the plasma the proportions of EPA and DHA were higher with the supplemented diet. The same authors also concluded that the maternal dietary α-linolenic acid content seems to be necessary for the deposition of n-3 LC-PUFA in foetal tissues.

Farmer and Petit [43] showed that feeding linseed or linseed oil can also bring about increases in DHA in the carcass and brain tissues of 1-d-old piglets. Bazinet, McMillan and Cunnane [62] reported that piglets from sows on a diet supplemented with α-linolenic acid had 54% more DHA in the liver and 24% more in the brain at 14-d old compared to the piglets from sows on a control diet. Similarly, Groot et al. [63], in a human study, reported that maternal PUFA supplementation during pregnancy increased the proportion of EPA two-fold in the plasma of newborns at birth. These studies confirm that PUFA supplementation can increase LC-PUFA status in the newborn, so it is essential to consider the lipid composition of foods such as milk and its derivatives in the diet of pregnant women, as it directly reflects on the FA profile of their offspring. Since pigs have a metabolism very similar to that of humans, the results of the present study show the great potential for pregnant women, and also for their newborn babies, of consuming cow’s milk enriched with PUFA.

## 5. Conclusions

In conclusion, our results indicate that the gilts supplemented with cow’s milk naturally enriched with n-3 and n-6 from 34 days of age until the end of lactation showed an effect on the FA profile of their offspring and themselves, it being important to note that in piglets there was a significant increase in EPA in the n-3 treatment. Regarding the females’ metabolism, there was a reduction in LDL for the n-3 and n-6 treatments, besides improving the performance of piglets with better average body weight in the lactation period from d 1–21.

## Figures and Tables

**Table 1:** The lipid fraction of milk from Holstein dairy cows fed diets supplemented linseed and soybean oil.

Fatty Acid Profile ^a^, g/100 g	Diets ^b^			SEM ^c^		*p*-Value ^d^	
	CON	LIN	SOY		Treatment	C1	C2
ΣSFA	66.89	56.60	56.52	1.441	<0.01	<0.01	0.969
ΣUSFA	33.05	43.35	43.39	1.438	<0.01	<0.01	0.986
SFA/USFA	2.12	1.36	1.34	0.107	<0.01	<0.01	<0.01
ΣMUFA	29.58	39.47	39.55	1.301	<0.01	<0.01	0.966
ΣPUFA	3.57	3.98	3.93	0.275	0.199	0.076	0.860
Σn-3	0.32	1.02	0.36	0.029	<0.01	<0.01	<0.01
Σn-6	2.48	2.25	2.88	0.208	0.004	0.589	0.001
n-6/n-3	7.92	2.72	8.26	0.504	<0.01	<0.01	<0.01
Cholesterol, g/100 mL	9.94	8.74	10.05	1.649	0.174	0.403	0.097

^a^ ΣSFA = Σ saturated fatty acids; ΣUSFA = Σ unsaturated fatty acids; SFA/USFA = Σ saturated/Σ unsaturated; ΣMUFA = Σ monounsaturated fatty acids; ΣPUFA = Σ polyunsaturated fatty acids; Σn-3 = sum of n-3 fatty acids; Σn-6 = n-6: sum of n-6 fatty acids; n-6/n-3 = Σ omega-6/Σ omega-3. ^b^ Cows fed with a Control diet (CON), supplemented with linseed oil (LIN) or soybean oil (SOY). ^c^ SEM, standard error of the mean. ^d^ C1, contrast CON vs. LIN+SOY; C2, contrast LIN vs. SOY.

**Table 2:** Composition of pre-initial, initial, grower and finisher diets, specific to each physiological phase of swine females.

Item	Pre-Initial	Initial	Grower	Finisher	Replacement	Gestation	Lactation
Ingredients, g/kg							
Ground corn	399.0	649.0	699.0	739.0	644.4	594.0	587.8
Soybean meal	200.0	300.0	280.0	240.0	240.8	140.0	265.1
Wheat bran	-	-	-	-	86.5	240.0	-
UNIMIX ^a^	400.0	50.0	20.0	20.0	25.0	25.0	30.0
L-lysine	-	-	-	-	-	-	10.0
DL-methionine	-	-	-	-	-	-	2.0
Sugar	-	-	-	-	-	-	100.0
Calcitic limestone	-	-	-	-	2.3	-	4.1
Mycofix	1.0	1.0	1.0	1.0	1.0	1.0	1.0
Chemical composition							
Dry matter, %	83.41	87.92	87.07	88.93	88.12	89.42	88.25
Ashes, %	6.69	5.43	4.80	3.70	4.54	5.23	5.62
Crude energy, cal/g	4347.0	4393.0	4419.0	4465.0	4411.5	4360.0	4376.0
Ether extract, %	4.14	1.71	2.41	1.71	1.37	1.40	1.56
Crude fiber, %	4.54	4.32	4.30	4.07	3.01	5.23	5.28
Crude protein, %	18.04	21.21	18.07	20.46	19.04	17.59	19.99
Calcium, %	0.90	0.80	0.63	0.51	0.82	0.81	0.94
Phosphorus, %	0.69	0.53	0.41	0.37	0.38	0.46	0.43

^a^ The premix used in each phase was based on Rostagno et al. [16].

**Table 3:** Fatty acid composition and lipid fraction of diets offered to swine females.

Fatty Acids ^a^, g/100 g	Pre-Initial	Initial	Grower	Finisher	Replacement	Gestation	Lactation
Lauric, C12:0	10.63	0.17	0.01	0.07	0.01	0.03	0.01
Myristic, C14:0	4.17	0.20	0.07	0.14	0.09	0.18	0.14
Palmitic, C16:0	18.24	21.31	16.19	20.52	17.92	17.72	17.47
Stearic, C18:0	5.04	3.58	2.70	2.62	2.62	2.48	2.68
Palmitoleic, C16:1c9	0.13	0.08	0.07	0.09	0.07	0.18	0.15
Elaidic, C18:1t	0.21	n.d. ^c^	n.d. ^c^	n.d. ^c^	n.d. ^c^	0.06	0.05
Oleic, C18:1c9	23.75	25.72	32.63	25.46	25.52	22.11	23.39
Vaccenic, C18:1c11	1.44	1.56	1.76	1.53	1.50	1.55	1.39
Linoleic, C18:2 n-6	28.94	43.40	43.36	46.01	48.70	51.52	50.41
α-Linolenic, C18:3 n-3	1.84	1.78	1.59	1.71	2.11	2.26	2.35
DHA, C22:06 n-3	n.d.^c^	0.06	0.04	0.03	n.d.^c^	0.07	0.06
Others ^b^	5.55	2.06	1.57	1.82	1.48	1.81	1.81
ΣSFA	42.96	26.73	19.86	24.43	21.45	21.33	21.32
ΣUSFA	56.30	72.55	79.41	74.80	77.89	77.67	77.74
ΣMUFA	26.19	27.97	35.14	27.82	27.74	24.75	25.74
ΣPUFA	30.77	45.24	44.99	47.75	50.81	53.87	52.84
Σn-3	1.84	1.84	1.63	1.74	2.11	2.32	2.41
Σn-6	28.94	43.40	43.36	46.01	48.70	51.52	50.41
SFA/USFA	0.76	0.37	0.25	0.33	0.28	0.27	0.27
n-6/n-3	15.76	23.58	26.57	26.38	23.10	22.19	20.92
Total	99.92	99.93	99.99	100.00	100.00	99.95	99.90

^a^ DHA: docosahexaenoic acid; ΣSFA = Σ saturated fatty acids; ΣUSFA = Σ unsaturated fatty acids; ΣMUFA = Σ monounsaturated fatty acids; ΣPUFA = Σ polyunsaturated fatty acids; Σ n-3 = Σ omega-3 fatty acids; Σ n-6 = Σ omega-6 fatty acids; SFA/USFA = Σ saturated/Σ unsaturated; n-6/n-3 = Σ omega-6/Σ omega-3. ^b^ Composed of the following fatty acids: C4:0, C6:0, C8:0, C10:0, C10:1, C11:0, C12:1, C13:0 iso, C13:0, C14:0 iso, C14:1c9, C15:0 iso, C15:0 anteiso, C15:0, C17:0 iso, C17:0, C17:1, C18:1 c12, C18:1 c13, C20:0, C20:1, C20:2, C22:0, C22:1n9, C23:0, C24:0, C24:1. ^c^ Not detectable.

**Table 4:** Concentrations of urea, total protein, total cholesterol, glucose, triglycerides, HDL, LDL and VLDL in the serum of sows on the first day of lactation.

Biochemical Parameters	Treatments ^a^			SEM ^b^	*p*-Value ^c^		
	Control	n-3	n-6		Treatment	C1	C2
Urea, mg/dL	23.70	28.22	24.88	1.689	0.841	<0.001	0.841
Total protein, g/dL	6.93	6.24	6.50	0.165	0.022	0.010	0.285
Total cholesterol, mg/dL	53.90	46.44	47.80	3.450	0.281	0.120	0.775
Glucose, mg/dL	70.60	77.22	74.55	3.610	0.478	0.266	0.636
Triglycerides, mg/dL	24.09	31.11	35.33	3.950	0.117	0.062	0.327
HDL, mg/dL	16.50	14.77	14.88	1.480	0.654	0.362	0.959
LDL, mg/dL	32.66	26.13	24.76	2.280	0.039	0.012	0.684
VLDL, mg/dL	4.70	6.22	7.11	0.758	0.094	0.043	0.427

^a^ Sows fed with a Control milk (Control), supplemented with cow’s milk enriched with n-3 or n-6. ^b^ SEM, standard error of the mean. ^c^ C1, contrast Control vs. n-3+n-6; C2, contrast n-3 vs. n-6.

**Table 5:** Fatty acid profile of the serum of sows on the first day of lactation.

Fatty Acids ^a^, µg/mL	Treatments ^b^			SEM ^c^	*p*-Value ^d^		
	Control	n-3	n-6		Treatment	C1	C2
Myristic, C14:0	0.47	0.63	0.54	0.378	0.652	0.562	0.568
Palmitic, C16:0	16.80	18.53	15.76	0.613	0.029	0.709	0.012
Stearic, C18:0	13.08	13.48	16.45	1.029	0.133	0.252	0.066
Elaidic, C18:1t	0.20	0.28	0.22	0.036	0.323	0.398	0.281
Palmitoleic, C16:1c9	0.99	1.54	1.27	0.281	0.431	0.351	0.482
Oleic, C18:1c9	22.65	22.86	25.62	1.702	0.461	0.541	0.253
Linoleic, C18:2 n-6	32.01	29.43	25.95	1.411	0.102	0.079	0.104
Dihomo-γ-linolenic, C20:3 n-6	0.51	0.65	0.68	0.125	0.708	0.426	0.832
ARA, C20:4 n-6	11.77	10.78	11.91	1.035	0.647	0.786	0.425
γ-Linolenic, C18:3 n-6	0.45	0.71	0.48	0.065	0.033	0.161	0.031
α-Linolenic, C18:3 n-3	0.58	0.54	0.50	0.104	0.899	0.715	0.750
EPA, C20:5 n-3	0.29	0.34	0.30	0.042	0.675	0.659	0.517
DHA, C22:6 n-3	0.20	0.22	0.31	0.045	0.304	0.335	0.177
ΣSFA	30.35	32.65	32.74	1.030	0.348	0.348	0.287
ΣUSFA	69.65	67.35	67.26	3.090	0.432	0.167	0.948
SFA/USFA	0.44	0.48	0.49	2.252	0.354	0.171	0.950
ΣMUFA	23.84	24.67	27.11	1.806	0.520	0.461	0.334
ΣPUFA	45.81	42.67	40.15	1.469	0.153	0.084	0.229
Σn-3	1.07	1.10	1.11	0.121	0.975	0.828	0.966
Σn-6	44.74	41.57	39.03	1.465	0.149	0.081	0.226
n-6/n-3	43.00	38.75	34.71	3.949	0.499	0.315	0.455
ARA/EPA	41.15	32.92	39.34	5.406	0.498	0.544	0.391

^a^ ARA: arachidonic acid; EPA: eicosapentaenoic acid; DHA: docosahexaenoic acid; ΣSFA = Σ saturated fatty acids; ΣUSFA = Σ unsaturated fatty acids; USFA/SFA: unsaturated/saturated fatty acids; ΣMUFA = Σ monounsaturated fatty acids; ΣPUFA = Σ polyunsaturated fatty acids; Σn-3 = sum of n-3 fatty acids; Σ n-6 = n-6: sum of n-6 fatty acids; n-6/n-3, ratio of total n-6 to total n-3 fatty acids; ARA/EPA: ratio of arachidonic acid to eicosapentaenoic acid. ^b^ Sows fed with a Control milk (Control), supplemented with cow’s milk enriched with n-3 or n-6. ^c^ SEM, standard error of the mean. ^d^ C1, contrast Control vs. n-3+n-6; C2, contrast n-3 vs. n-6.

**Table 6:** Effects of enriched milk supplementation on colostrum composition of lactating sows.

Chemical Composition (g/100 g)	Treatments ^a^			SEM ^b^	*p*-Value ^c^		
	Control	n-3	n-6		Treatment	C1	C2
Colostrum							
Fat	-	-	-	-	-	-	-
Protein	6.29	5.96	5.69	0.213	0.157	0.075	0.413
Lactose	9.46	8.97	8.61	0.320	0.184	0.087	0.454
Solids-not-fat	17.18	16.41	15.60	0.642	0.263	0.130	0.424
Milk							
Fat	9.53	9.88	8.39	0.968	0.551	0.745	0.302
Protein	3.84	3.90	3.76	0.085	0.507	0.891	0.252
Lactose	5.74	5.82	5.61	0.122	0.500	0.901	0.246
Solids-not-fat	10.42	10.58	10.20	0.223	0.500	0.891	0.247

^a^ Sows fed with a Control milk (Control), supplemented with cow’s milk enriched with n-3 or n-6. ^b^ SEM, standard error of mean. ^c^ C1, contrast Control vs. n-3+n-6; C2, contrast n-3 vs. n-6.

**Table 7:** Effect of supplementation of milk enriched with n-3 and n-6 on the fatty acid profile of sows’ colostrum.

Fatty Acid Profile ^a^, g/100 g	Treatments ^b^			SEM ^c^	*p*-Value ^d^		
	Control	n-3	n-6		Treatment	C1	C2
Caproic, C6:0	0.011	0.000 *	0.000 *	0.003	0.309	0.133	1.000
Caprylic, C8:0	0.000 *	0.000 *	0.000 *	0.000	0.619	0.337	1.000
Capric, C11:0	0.010	0.009	0.008	0.001	0.834	0.623	0.743
Hendecanoic, C11:0	0.001	0.001	0.000 *	0.000	0.844	0.617	0.781
Lauric, C12:0	0.062	0.053	0.047	0.005	0.453	0.265	0.579
Isotridecyl, C13:0 iso	0.005	0.000 *	0.000*	0.002	0.400	0.341	0.342
Anteisotridecylic, C13:0 anteiso	0.018	0.000 *	0.000*	0.003	0.092	0.032	1.000
Tridecylic, C13:0	0.004	0.003	0.001	0.001	0.085	0.131	0.086
Isomyristic, C14:0 iso	0.012	0.009	0.008	0.001	0.124	0.047	0.802
Myristic, C14:0	2.424	2.516	2.216	0.110	0.556	0.813	0.300
Isopentadecylic, C15:0 iso	0.047	0.051	0.082	0.005	0.004	0.024	0.005
Anteisopentadecylic, C15:0 anteiso	0.076	0.072	0.066	0.003	0.498	0.336	0.504
Pentadecylic, C15:0	0.249	0.268	0.235	0.008	0.271	0.870	0.115
Isopalmitic, C16:0 iso	0.033	0.025	0.031	0.002	0.351	0.334	0.281
Palmitic, C16:0	25.550	24.526	25.360	0.295	0.346	0.346	0.266
Isomargaric, C17:0 iso	0.033	0.022	0.015	0.002	0.001	0.001	0.084
Margaric, C17:0	0.385	0.421	0.404	0.013	0.573	0.365	0.607
Stearic, C18:0	5.347	5.160	5.529	0.151	0.641	0.995	0.356
Arachidic, C20:0	0.094	0.082	0.091	0.003	0.344	0.300	0.303
Behenic, C22:0	0.057	0.054	0.054	0.003	0.906	0.664	0.981
Tricosylic, C23:0	0.015	0.016	0.011	0.001	0.234	0.615	0.108
Lignoceric, C24:0	0.069	0.061	0.078	0.006	0.536	0.994	0.274
Lauroleic, C12:1	0.000*	0.001	0.001	0.000	0.397	0.187	0.834
Myristoleic, C14:1c9	0.050	0.050	0.038	0.004	0.357	0.468	0.219
Palmitoleic, C16:1c9	2.656	2.377	2.502	0.100	0.420	0.275	0.631
cis-10-Heptadecenoic, C17:1c10	0.263	0.305	0.314	0.010	0.091	0.034	0.683
Elaidic, C18:1t	0.494	0.704	0.713	0.038	0.016	0.005	0.900
Oleic, C18:1c9	32.282	31.780	32.843	0.547	0.759	0.981	0.467
Vaccenic, C18:1c11	3.859	3.722	3.882	0.091	0.770	0.785	0.513
cis-12-Octadecenoic, C18:1c12	0.380	0.379	0.356	0.010	0.562	0.570	0.370
cis-13-Octadecenoic, C18:1c13	0.210	0.175	0.201	0.007	0.130	0.145	0.146
trans-16-Octadecenoic, C18:1t16	0.035	0.037	0.040	0.003	0.696	0.521	0.587
cis-15-Octadecenoic, C18:1c15	0.008	0.068	0.010	0.008	<0.001	<0.001	<0.001
Gondoic, C20:1	0.293	0.273	0.271	0.008	0.486	0.241	0.921
Erucic, C22:1	0.098	0.127	0.121	0.006	0.127	0.050	0.663
Nervonic, C24:1	0.119	0.139	0.151	0.009	0.364	0.196	0.576
Linoleic, C18:2 n-6	21.612	23.177	21.260	0.535	0.319	0.596	0.162
γ-Linolenic, 18:3 n-6	0.206	0.198	0.208	0.011	0.938	0.918	0.737
α-Linolenic, 18:3 n-3	0.783	1.081	0.827	0.042	0.001	0.007	0.001
Octadeca-9,11-dienoic, C18:2c9t11	0.073	0.092	0.073	0.006	0.321	0.418	0.205
11cis-14cis-Eicosadienoic, C20:2	0.327	0.282	0.288	0.014	0.400	0.188	0.863
Dihomo-γ-linolenic, 20:3 n-6	0.252	0.217	0.238	0.011	0.431	0.299	0.442
Dihomo-α-linolenic, 20:3 n-3	0.043	0.038	0.035	0.003	0.564	0.330	0.686
ARA, C20:4 n-6	1.048	0.998	0.976	0.038	0.762	0.489	0.833
Docosa-13,16-dienoic, C22:2c13c16	0.031	0.030	0.029	0.002	0.973	0.757	0.901
EPA, C20:5 n-3	0.028	0.032	0.019	0.002	0.052	0.531	0.020
Clupadonic, C22:5	0.296	0.308	0.308	0.018	0.960	0.780	1.000
DHA, C22:6 n-3	0.074	0.056	0.063	0.008	0.605	0.348	0.896

^a^ ARA: arachidonic acid; EPA: eicosapentaenoic acid; DHA: docosahexaenoic acid. ^b^ Sows fed with a Control milk (Control), supplemented with cow’s milk enriched with n-3 or n-6; 0.000 *: Values lower than 10^−6^ g/100 g. ^c^ SEM, standard error of the mean. ^d^ C1, contrast Control vs. n-3+n-6; C2, contrast n-3 vs. n-6.

**Table 8:** Effect of supplementation of milk enriched with n-3 and n-6 on the quality of the lipid fraction of sows’ colostrum.

Item ^a^	Treatments ^b^				*p*-Value ^d^		
	Control	n-3	n-6	SEM ^c^	Treatment	C1	C2
ΣSFA	34.50	33.35	34.24	0.341	0.380	0.345	0.308
ΣUSFA	65.52	66.64	65.77	0.338	0.390	0.357	0.310
ΣMUFA	40.75	40.14	41.44	0.648	0.742	0.977	0.449
ΣPUFA	24.77	26.51	24.32	0.611	0.328	0.624	0.164
Σn-3	0.93	1.21	0.94	0.045	0.005	0.045	0.005
Σn-6	23.12	24.59	22.68	0.556	0.367	0.667	0.184
SFA/USFA	0.53	0.50	0.52	0.008	0.294	0.344	0.194
n-6/n-3	25.18	20.40	24.10	0.714	0.001	0.016	0.003

^a^ ΣSFA = Σ saturated fatty acids; ΣUSFA = Σ unsaturated fatty acids; ΣMUFA = Σ monounsaturated fatty acids; ΣPUFA = Σ polyunsaturated fatty acids; Σn-3 = sum of n-3 fatty acids; Σn-6 = n-6: sum of n-6 fatty acids; SFA/USFA = Σ saturated/Σ unsaturated; n-6/n-3 = Σ omega-6/Σ omega-3. ^b^ Sows fed with a Control milk (Control), supplemented with cow’s milk enriched with n-3 or n-6. ^c^ SEM, standard error of the mean. ^d^ C1, contrast Control vs. n-3+n-6; C2, contrast n-3 vs. n-6.

**Table 9:** Effect of supplementation of milk enriched with n-3 and n-6 on the fatty acid profile of sows’ milk sampled at d 14 of lactation.

Fatty Acid Profile ^a^, g/100 g	Treatments ^b^				*p*-Value ^d^		
	Control	n-3	n-6	SEM ^c^	Treatment	C1	C2
Butiric, C4:0	0.025	0.027	0.032	0.003	0.627	0.448	0.561
Caproic, C6:0	0.019	0.022	0.037	0.004	0.119	0.196	0.098
Caprylic, C8:0	0.031	0.040	0.041	0.004	0.561	0.293	0.956
Capric, C11:0	0.189	0.250	0.238	0.027	0.659	0.379	0.862
Caproleic, C10:1c9	0.016	0.031	0.028	0.004	0.312	0.135	0.775
Hendecanoic, C11:0	0.002	0.001	0.001	0.000	0.384	0.180	0.817
Lauric, C12:0	0.307	0.334	0.320	0.023	0.906	0.710	0.818
Isotridecyl, C13:0 iso	0.003	0.001	0.002	0.001	0.655	0.493	0.552
Anteisotridecylic, C13:0 anteiso	0.002	0.000 *	0.000 *	0.001	0.397	0.183	1.000
Lauroleic, C12:1	0.004	0.006	0.005	0.001	0.762	0.513	0.605
Tridecylic, C13:0	0.004	0.004	0.003	0.001	0.833	0.680	0.668
Isomyristic, C14:0 isso	0.004	0.005	0.003	0.001	0.235	0.262	0.230
Myristic, C14:0	3.218	3.650	3.570	0.196	0.666	0.384	0.876
Isopentadecylic, C15:0 iso	0.076	0.031	0.048	0.011	0.253	0.129	0.523
Anteisopentadecylic, C15:0 anteiso	0.041	0.038	0.041	0.002	0.874	0.777	0.672
Myristoleic, C14:1c9	0.203	0.304	0.262	0.034	0.494	0.249	0.595
Pentadecylic, C15:0	0.134	0.122	0.120	0.007	0.722	0.431	0.939
Isopalmitic, C16:0 iso	0.015	0.017	0.017	0.002	0.798	0.751	0.563
Palmitic, C16:0	27.363	28.150	28.129	0.877	0.927	0.704	0.993
Isomargaric, C17:0 iso	0.112	0.017	0.020	0.032	0.540	0.739	0.281
Palmitoleic, C16:1c9	7.722	9.438	9.709	0.881	0.642	0.360	0.906
Margaric, C17:0	0.279	0.223	0.229	0.016	0.292	0.126	0.879
cis-10-Heptadecenoic, C17:1c10	0.380	0.404	0.350	0.015	0.355	0.913	0.159
Stearic, C18:0	4.761	3.727	3.935	0.319	0.405	0.195	0.795
Elaidic, C18:1t	0.382	0.365	0.450	0.023	0.292	0.603	0.144
Oleic, C18:1c9	36.788	36.110	35.053	1.300	0.878	0.689	0.761
Vaccenic, C18:1c11	3.637	3.633	3.724	0.135	0.959	0.896	0.803
cis-12-Octadecenoic, C18:1c12	0.362	0.386	0.375	0.032	0.959	0.802	0.897
cis-13-Octadecenoic, C18:1c13	0.199	0.173	0.196	0.011	0.480	0.569	0.325
trans-16-Octadecenoic, C18:1t16	0.032	0.029	0.025	0.002	0.510	0.352	0.498
cis-15-Octadecenoic, C18:1c15	0.009	0.021	0.006	0.002	0.004	0.075	0.001
Linoleic, C18:2 n-6	11.602	10.571	11.062	0.249	0.254	0.148	0.420
Arachidic, C20:0	0.067	0.056	0.064	0.003	0.275	0.195	0.230
γ-Linolenic, 18:3 n-6	0.102	0.071	0.094	0.010	0.260	0.138	0.265
α-Linolenic, 18:3 n-3	0.470	0.485	0.475	0.010	0.849	0.667	0.714
Gondoic, C20:1	0.312	0.284	0.266	0.026	0.804	0.549	0.806
Octadeca-9,11-dienoic, C18:2c9t11	0.058	0.057	0.063	0.006	0.914	0.870	0.702
11cis-14cis-Eicosadienoic, C20:2	0.178	0.156	0.155	0.018	0.855	0.584	0.983
Dihomo-γ-linolenic, 20:3 n-6	0.088	0.070	0.068	0.005	0.226	0.093	0.845
Behenic, C22:0	0.022	0.025	0.025	0.002	0.769	0.478	1.000
Dihomo-α-linolenic, 20:3 n-3	0.021	0.019	0.015	0.003	0.574	0.521	0.558
ARA, C20:4 n-6	0.420	0.366	0.459	0.019	0.036	0.900	0.012
Erucic, C22:1	0.087	0.079	0.091	0.005	0.603	0.859	0.332
Tricosylic, C23:0	0.007	0.006	0.004	0.001	0.542	0.435	0.440
Docosa-13,16-dienoic, C22:2c13c16	0.012	0.011	0.014	0.001	0.716	0.810	0.444
EPA, C20:5 n-3	0.015	0.016	0.015	0.002	0.965	0.896	0.820
Lignoceric, C24:0	0.022	0.020	0.016	0.004	0.783	0.628	0.647
Nervonic, C24:1	0.079	0.054	0.063	0.009	0.552	0.318	0.694
Clupadonic, C22:5	0.107	0.091	0.089	0.005	0.268	0.117	0.759
DHA, C22:6 n-3	0.017	0.007	0.037	0.007	0.073	0.498	0.133

^a^ ARA: arachidonic acid; EPA: eicosapentaenoic acid; DHA: docosahexaenoic acid. ^b^ Sows fed with a Control milk (Control), supplemented with cow’s milk enriched with n-3 or n-6; 0.000 *: Values lower than 10^−6^ g/100 g. ^c^ SEM, standard error of the mean. ^d^ C1, contrast Control vs. n-3+n-6; C2, contrast n-3 vs. n-6.

**Table 10:** Effect of supplementation of milk enriched with n-3 and n-6 on the quality of the lipid fraction of sows’ milk.

Item ^a^	Treatments ^b^			SEM ^c^	*p*-Value ^d^		
	Control	n-3	n-6		Treatment	C1	C2
ΣSFA	36.70	36.77	36.90	0.951	0.997	0.954	0.960
ΣUSFA	63.30	63.23	63.15	0.950	0.998	0.961	0.973
ΣMUFA	50.21	51.32	50.60	0.869	0.887	0.711	0.759
ΣPUFA	13.09	11.92	12.55	0.287	0.267	0.172	0.376
Σn-3	0.52	0.53	0.54	0.011	0.779	0.665	0.586
Σn-6	12.21	11.08	11.68	0.264	0.226	0.146	0.347
SFA/USFA	0.59	0.59	0.59	0.023	0.999	0.992	0.968
n-6/n-3	23.41	21.04	21.57	0.452	0.069	0.026	0.594

^a^ ΣSFA = Σ saturated fatty acids; ΣUSFA = Σ unsaturated fatty acids; ΣMUFA = Σ monounsaturated fatty acids; ΣPUFA = Σ polyunsaturated fatty acids; Σn-3 = sum of n-3 fatty acids; Σn-6 = n-6: sum of n-6 fatty acids; SFA/USFA = Σ saturated/Σ unsaturated; n-6/n-3 = Σ omega-6/Σ omega-3. ^b^ Sows fed with a Control milk (Control), supplemented with cow’s milk enriched with n-3 or n-6. ^c^ SEM, standard error of the mean. ^d^ C1, contrast Control vs. n-3+n-6; C2, contrast n-3 vs. n-6.

**Table 11:** Sow’ reproduction traits and piglet’s performance.

Trait	Treatments ^a^			SEM ^b^	*p*-Value ^c^				
	Control	n-3	n-6		Treatment	Time	Diet*Time	C1	C2
Results of sows									
Number of piglets per litter, n									
Total born	12.33	13.67	13.00	1.066	0.670	-	-	0.447	0.666
Born alive	11.33	13.00	11.75	0.659	0.201	-	-	0.202	0.202
Stillborn	0.22	0.44	0.75	0.186	0.327	-	-	0.232	0.426
Mummified	0.22	1.00	0.50	1.691	0.144	-	-	0.146	0.261
Time to first suckle	10.99	14.31	18.50	1.028	<0.001	-	-	<0.001	<0.001
Piglets body weight, kg									
1 d	1.33	1.09	1.23	0.029	<0.001	-	-	<0.001	<0.001
21 d	4.69	4.25	4.72	0.152	0.060	-	-	0.261	0.035
1 to 21 d	2.79	2.91	2.95	0.040	0.013	<0.001	0.117	0.004	0.460
Piglets average daily gain, kg/day									
1 to 7 d	0.14	0.13	0.15	0.008	0.277	-	-	-	-
7 to 14 d	0.16	0.19	0.19	0.008	0.015	-	-	0.003	0.552
14 to 21 d	0.16	0.16	0.15	0.008	0.739	-	-	-	-
1 to 21 d	0.15	0.16	0.16	0.005	0.649	<0.001	0.023	0.661	0.554

^a^ Sows fed with a Control milk (Control), supplemented with cow’s milk enriched with n-3 or n-6. ^b^ SEM, standard error of the mean. ^c^ C1, contrast Control vs. n-3+n-6; C2, contrast n-3 vs. n-6.

**Table 12:** Piglet serum fatty acids profile at 1 and 14 days age.

Fatty acids (µg/mL) ^a^	Treatments ^b^						SEM ^c^	*p*-Value ^d^				
	Control		n-3		n-6							
	D1	D14	D1	D14	D1	D14		Treatment	Time	Treat*Time	C1	C2
Myristic, C14:0	1.54	1.12	1.85	1.14	1.81	0.96	0.215	0.724	0.004	0.675	0.543	0.603
Palmitic, C16:0	23.90	22.80	25.58	24.47	24.46	22.94	1.071	0.357	0.149	0.975	0.346	0.273
Stearic, C18:0	13.98	10.88	13.54	10.83	13.41	11.44	0.632	0.915	<0.001	0.691	0.830	0.721
Elaidic, 18:1t	0.61	0.26	0.40	0.21	0.51	0.30	0.073	0.299	0.002	0.559	0.324	0.244
Palmitoleic, C16:1c9	5.59	4.20	6.13	4.54	5.67	4.29	0.675	0.833	0.010	0.977	0.701	0.644
Oleic, C18:1c9	25.25	23.82	23.98	21.26	22.89	21.12	1.87	0.234	0.036	0.799	0.104	0.675
Linoleic, C18:2 n-6	5.21	21.96	5.19	23.23	5.35	23.81	0.747	0.527	<0.001	0.642	0.297	0.666
Dihomo-γ-linolenic acid, C20:3 n-6	0.98	0.52	0.97	0.69	0.68	0.67	0.090	0.293	0.005	0.095	0.979	0.124
ARA, C20:4 n-6	18.18	11.19	17.68	11.25	19.35	11.62	0.753	0.419	<0.001	0.709	0.686	0.217
γ-Linolenic, C18:3 n-6	0.41	0.65	0.44	0.49	0.49	0.41	0.074	0.632	0.183	0.111	0.361	0.855
α-Linolenic, C18:3 n-3	0.05	0.39	0.08	0.41	0.04	0.40	0.037	0.780	<0.001	0.885	0.721	0.549
EPA, C20:5 n-3	0.40	0.12	0.61	0.19	0.36	0.14	0.051	0.016	<0.001	0.184	0.133	0.010
DHA, C22:6 n-3	3.82	1.60	4.00	1.75	4.47	1.83	0.282	0.385	<0.001	0.693	0.287	0.383
ΣSFA	39.48	34.90	40.87	36.34	39.78	35.36	0.925	0.384	<0.001	0.996	0.335	0.320
ΣUSFA	60.52	65.10	59.13	63.66	60.22	64.64	0.925	0.384	<0.001	0.996	0.335	0.320
USFA/SFA	65.33	53.72	69.47	57.16	66.25	54.71	2.575	0.409	<0.001	0.981	0.360	0.330
ΣMUFA	31.32	28.26	30.55	26.05	29.07	25.71	1.425	0.326	0.006	0.841	0.175	0.558
ΣPUFA	29.36	36.83	28.59	37.62	31.12	38.93	1.582	0.406	<0.001	0.865	0.500	0.248
Σn-3	4.26	2.11	4.68	2.33	4.89	2.37	0.260	0.319	<0.001	0.759	0.156	0.659
Σn-6	25.10	34.71	23.91	35.29	26.22	36.55	1.446	0.429	<0.001	0.830	0.646	0.229
n-6/n-3	6.03	17.15	5.34	15.75	5.26	15.83	1.254	0.696	<0.001	0.951	0.412	0.997
ARA/EPA	46.75	86.62	33.92	67.48	52.73	85.28	8.411	0.092	<0.001	0.909	0.364	0.045

^a^ ARA: arachidonic acid; EPA: eicosapentaenoic acid; DHA: docosahexaenoic acid; ΣSFA = Σ saturated fatty acids; ΣUSFA = Σ unsaturated fatty acids; USFA/SFA: unsaturated/saturated fatty acids; ΣMUFA = Σ monounsaturated fatty acids; ΣPUFA = Σ polyunsaturated fatty acids; Σ n-3 = sum of n-3 fatty acids; Σ n-6 = n-6: sum of n-6 fatty acids; n-6/n-3, ratio of total n-6 to total n-3 fatty acids; ARA/EPA: ratio of arachidonic acid to eicosapentaenoic acid. ^b^ Sows fed with a Control milk (C), supplemented with cow’s milk enriched with n-3 or n-6. ^c^ SEM, standard error of the mean. ^d^ C1, contrast Control vs. n-3+n-6; C2, contrast n-3 vs. n-6.

## Supplementary Materials

The following are available online at https://www.mdpi.com/article/10.3390/nu13061942/s1, Figure S1: Scheme of the physiological phases of the swine females and consumption of enriched milk in each phase of the experiment, Table S1: Sow’s performance from 1st to 21st day of lactation.

## Abbreviations and Acronyms

ARA	arachidonic acid (20:4 n-6)
ADG	average daily gain
AI	artificial insemination
AA	arachidonic acid (20:4 n-6)
BW	body weight
DHA	docosahexaenoic acid (22:6 n-3)
DPAn-3	docosapentaenoic acid (22:5 n-3)
EPA	eicosapentaenoic acid (20:5 n-3)
FA	fatty acid(s)
FAME	fatty acid methyl ester(s)
HDL	high-density lipoprotein
LC-PUFA	long-chain polyunsaturated fatty acid(s)
LDL	low-density lipoprotein
mRNA	messenger RNA
MUFA	monounsaturated fatty acid(s)
PUFA	polyunsaturated fatty acid(s)
TG	triglicerydes
VLDL	very-low-density lipoprotein
