# Supplementary material for: Maternal Supplementation with Cow’s Milk Naturally Enriched with PUFA Alters the Metabolism of Sows and the Fatty Acid Profile of the Offspring
Source: Nutrients. 2021 Jun 5;13(6):1942. doi: 10.3390/nu13061942 (PMC8228345; doi:10.3390/nu13061942)
Supplement: Supplementary file 1 [file nutrients-13-01942-s001.zip › nutrients-1176856-supplementary.pdf]

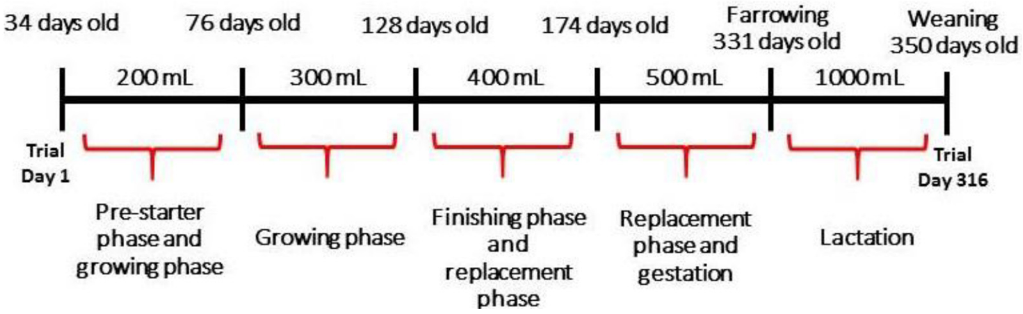

Figure S1. Scheme of the physiological phases of the swine females, and consumption of enriched milk in each phase of the experiment.

The figure represents the ages and the respective phases in which the increase in supplemented milk occurred, as well as the quantities supplied.

Table S1. Sow's performance from 1st to 21st day of lactation.

			Treatments[a]		SEM[b]			*P-value*[c]	
Control		n-3	n-6	Treatment	Time	Diet*Time		C1	C2
Average weight, kg	179.29	183.29	180.62	1.930	0.309	<0.0001	0.470	0.245	0.339
Weight D1Ld, kg	188.35	192.06	184.75	4.872	0.594	-	-	0.993	0.312
Weight D21Le, kg	170.61	179.61	170.11	4.120	0.207	-	-	0.408	0.116
D21 – D1, kg	-18.69	-10.98	-16.36	3.520	0.284	-	-	0.246	0.299
Average thickness of back-fat, mm	16.80	17.33	16.63	0.434	0.497	0.846	0.554	0.729	0.266
Thickness of back-fat D1L, mm	17.03	16.90	17.00	0.521	0.982	-	-	0.903	0.891
Thickness of back-fat D21L, mm	16.56	17.79	16.29	0.640	0.243	-	-	0.535	0.125
D21 – D1, mm	-0.51	0.87	-0.74	0.765	0.275	-	-	0.525	0.149

[a] Sows fed with a Control milk (Control), supplemented with cow's milk enriched with n-3 or n-6. [b]SEM, standard error of the mean. [c]C1, contrast Control vs n-3+n-6; C2, contrast n-3 vs n-6. [d]D1L: 1st day of lactation. [e]D21L: 21st day of lactation.
